# Expression Levels of Inflammatory and Oxidative Stress-Related Genes in Skin Biopsies and Their Association with Pityriasis Alba

**DOI:** 10.3390/medicina56070359

**Published:** 2020-07-17

**Authors:** Margarita L. Martinez-Fierro, Griselda A. Cabral-Pacheco, Idalia Garza-Veloz, Andrés E. Campuzano-García, Alma P. Díaz-Alonso, Virginia Flores-Morales, Iram P. Rodriguez-Sanchez, Ivan Delgado-Enciso, Jorge Rios-Jasso

**Affiliations:** 1 Molecular Medicine Laboratory, Doctorado en Ciencias con Orientación en Medicina Molecular, Unidad Académica de Medicina Humana y C.S., Universidad Autónoma de Zacatecas, Zacatecas 98160, Mexico; gris_elda_ai91@hotmail.com (G.A.C.-P.); patriciaalonsodd@gmail.com (A.P.D.-A.); nietzx72@gmail.com (J.R.-J.); 2 Hospital General Zacatecas “Luz González Cosío”, Servicios de Salud de Zacatecas, Zacatecas 98160, Mexico; campuzano3@hotmail.com; 3 Laboratorio de Síntesis Asimétrica y Bioenergética (LSAyB), Doctorado en Ciencias con Orientación en Medicina Molecular, Unidad Académica de Medicina Humana y C.S., Universidad Autónoma de Zacatecas, Zacatecas 98160, Mexico; virginia.flores@uaz.edu.mx; 4 Laboratorio de Fisiología Molecular y Estructural, Facultad de Ciencias Biológicas, Universidad Autónoma de Nuevo León, Monterrey 66455, Mexico; iramrodriguez@gmail.com; 5 School of Medicine, University of Colima, and Cancerology State Institute, Colima State Health Services, Colima 28040, Mexico; ivan_delgado_enciso@ucol.mx

**Keywords:** Pityriasis Alba, gene expression, oxidative stress, inflammatory stress

## Abstract

*Background and objectives*: Pytiriasis alba (PA) is a common skin disorder which affects 80% of children between six and 16 years. The etiology of PA is unclear, but hypo-pigmented patches in photo-exposed zones characterize the disease. Because the high ultraviolet exposition of the skin promotes an acute inflammatory response and an increase of oxidative stress (OS), this study aimed to evaluate the expression levels of inflammatory and OS-related genes in skin biopsies, and their association with PA. *Materials and Methods*: A cross-sectional study was carried out. Skin biopsies of the lesion sites and healthy skin (controls) from 16 children with PA were evaluated. The tissue expression of IL-4, IL-6, IL-17A, TNFα, INFγ, IL-1β, SOD1, and HMOX1 was analyzed by qRT-PCR, using SYBR Green and glyceraldehyde-3-phosphate dehydrogenase gene as the endogenous control. *Results*: There were differences in the ΔCq values of HMOX1, SOD1, IL-6, and IFNγ between tissue with lesions and healthy skin (*p* < 0.05). Compared with healthy skin, IL-6, IFNγ, HMOX1, and SOD1 were predominantly under-expressed in the lesion sites. However, 25% of skin biopsies with lesions showed over-expression of these four genes. Positive correlations between the expression of IL-6 and HMOX1, SOD1, and IFNγ (*p* < 0.05) were also observed. *Conclusions*: Our results suggest the presence of molecular stages of PA, defined according to the over-expression (first stage) or under-expression (second stage) of the HMOX1, SOD1, IL-6, and IFNγ genes in abnormal skin tissue. These findings may have implications for the selection of treatment for PA-related lesions.

## 1. Introduction

Pityriasis alba (PA) is a self-limited benign skin disease that occurs most frequently in childhood [1]. PA commonly occurs in patients between 6 and 16 years of age, and affects 80% of children living in rural areas of developing countries [2,3]. Risk factors of PA are closely associated with those of atopic dermatitis, and include exposure to sunlight, low socioeconomic strata, nutritional deficiencies, and iron deficiency anemia, among others [4]. Furthermore, PA appears earlier in patients with skin phototype IV and V [2].

Clinically, PA is characterized by hypochromic scaly macules that appear more frequently in areas of skin exposed to sunlight [5]. Macules are oval with defined edges, and are up to 4 cm in diameter. Likewise, hypopigmented lesions of PA can recur [2]. Usually, two to three hypochromic lesions occur on the cheeks, around the mouth, around the eyes, and on the front and back of the forearms, but the rest of the skin on the body is not exempt [6]. In adults, the characteristic macules of PA may occur on the lower portion of the body trunk in a disseminated form. Histological studies have shown hyperkeratosis, spongiosis, acanthosis, and lymphocytic infiltration [7]. Three stages of the disease have been described. The early-stage characterized by well-delimited macules less than 2 cm in diameter, with erythema and follicular papules with points. In the intermediate stage the lesions increase in size up to 5 cm, and there is desquamatory hypopigmentation with tiny pointy papules compared to the previous stage. Finally, in the late stage, there are patches of 2–5 cm in diameter, hypopigmented, with irregular edges, with fine desquamation and without follicular papules [6]. In addition, rare variants have been described in adults, where the lesions are hyperchromic and diffuse [2].

The etiology of PA is unknown and there are currently few studies focused on this condition. The most acceptable explanation for the physiopathology of PA proposes an insult in the epidermis caused by ultraviolet (UV) irradiation [8]. Inflammatory cytokines, produced by keratinocytes or other immune cells, are increased after UV exposition and are closely related to changes in pigmentation in other skin disorders [8,9,10]. These molecules include interleukin-18 (IL-18) and interferon gamma (IFN-γ), among others [11,12,13,14]. Accordingly, and because the type IV hypersensitivity mechanism that causes post-inflammatory skin depigmentation has been previously suggested to occur in PA [2], in this study, the levels of inflammatory (*IL-4*, *IL-6*, *IL-1β*, *IL-17A*, tumor necrosis factor-alpha, and *INFγ*) and oxidative stress-related genes (superoxide dismutase 1 and heme oxygenase 1) were quantified in skin biopsies, and their association with PA was evaluated.

## 2. Materials and Methods

### 2.1. Study Population

We performed a cross-sectional study in Zacatecas, Mexico. It was conducted according to the guidelines laid down in the Declaration of Helsinki, and the protocol was approved by the Ethics Committee of the General Hospital “Luz Gonzalez Cosío” (Approval ID: 02020/2019, Date: 8 July 2019). The participants were recruited in the Dermatology service of General Hospital “Luz González Cosío” from the pediatric population referred for medical consultation for possible PA. The confirmation of the study population was done as follows: (1) Detailed information related to the protocol was provided to each participant, and his/her parent or guardian, and written informed consent and assent were obtained. All the participants who provided signed informed consent/assent underwent a physical examination and completed a questionnaire about the risk factors for PA. (2) Then, children with PA were identified for the study according to the guidelines of The British Association of Dermatologists and previous reports of PA differential diagnosis [15,16]. The study exclusion criteria involved the existence of other conditions, such as other hypopigmentation-related diseases, vitiligo, pityriasis veriscolor, treatment with steroids and/or photoprotection in the last three months, recent strong sun exposure such as outside sport activities, previous diagnosis of atopic dermatitis, and/or positive test by Wood lamp for other pathologies. Accordingly, 16 patients were included in the final study.

### 2.2. Biological Samples and Clinical Data

Two 3 mm punch biopsies, 10 mm apart, were taken from the lesion (child’s face) under local anesthesia. Each participant also donated a healthy skin biopsy (control) from the arm area, located in its internal region where there was no sun exposure. Tissue biopsies were preserved in RNA later solution (Life Technologies, Carlsbad, CA, USA) and stored at −80 °C until use. Additional clinical and laboratory data were obtained from the clinical records.

### 2.3. RNA Isolation and cDNA Synthesis

Total RNA was isolated from the skin biopsies using mechanical homogenization, using a Qiagen RNeasy^®^ Mini Kit (Qiagen, West Sussex, UK) according to the manufacturer’s protocol. The RNA concentration and its quality were determined by measurement of the optical density at 260 nm and the ratio 260/280, respectively, using a NanoDrop 2000 Spectrophotometer (Thermo Fisher Scientific, Wilmington, DE, USA). Complementary DNA (cDNA) was synthesized from 1.0 μg of total RNA in a final volume of 20 μL, using a High Capacity cDNA Reverse Transcription Kit (Thermo Fisher Scientific, Wilmington, DE, USA) and random hexamers, according to the manufacturer’s instructions. The final cDNA concentration was measured, and the samples were stored at −20 °C until use.

### 2.4. Gene Selection and Primer Design

Eight genes representing inflammatory signaling pathways and oxidative stress-related genes were selected for this study, including Interleukin (IL) 4 (*IL-4*), *IL-6*, *IL-17A*, *IL-1β*, Tumor necrosis factor-alpha (*TNFα*), interferon gamma (*INFγ*), superoxide dismutase 1 (*SOD1*), and heme oxygenase 1 (*HMOX1*). The glyceraldehyde-3-phosphate dehydrogenase (*GAPDH*) gene was selected as the internal control. Gene-specific primers for quantitative real-time polymerase chain reaction (qRT-PCR) assay were designed, and provided by T4OLIGO^®^ (T4OLIGO, Irapuato, GTO, Mexico). Sequences of forward and reverse primers and product sizes for all the evaluated genes are listed in Table 1.

### 2.5. Quantitative Real-Time Polymerase Chain Reaction

All the qRT- PCR experiments were conducted using a StepOne Plus Real-Time PCR System (Applied Biosystems, Foster City, CA, USA) in 96-well PCR plates. Each qRT-PCR reaction was performed in a 20 μL reaction mix containing 15 ng of template cDNA, 1X SYBR Green PCR Master Mix (Applied Biosystems, Foster City, CA, USA), and 500 nM of each primer. All samples were analyzed in duplicate, and each experiment included two non-template controls to detect any template contamination. Melting curve analysis was done to confirm the specificity of amplification and the lack of primer dimers. The thermal cycle program consisted of an initial denaturation at 95 °C for 10 min, followed by an amplification step for 40 cycles of 15 s at 95 °C and 1 min at 60 °C. The results were obtained using the 2^−ΔΔCq^ method [17], and delta Cq (ΔCq) values obtained for the different genes were compared using *GAPDH* as a reference.

### 2.6. Statistical Analysis

Categorical variables were described as frequency and percentages, and continuous variables were described using mean and median values. The normality of data distribution was evaluated using the Kolmogorov–Smirnov test. Simple comparisons of ΔCq values and/or relative expression levels between skin with lesions and healthy skin (control) were determined by Student’s *t*-test when the data were normally distributed; otherwise, the Mann–Whitney test was used. Pearson’s correlation analysis was done to determine whether there were correlations between the expression tissue levels of the genes of interest and clinical variables. Statistical analysis was carried out with the Sigma Plot version 12.0-statistic software package (Systat Software Inc., San Jose, CA, USA). *p*-values < 0.05 were considered significant.

## 3. Results

The study group included 16 patients between 6–11 years of age. Table 2 shows the general characteristics of the study population. Seventy-five percent of the cases were male, and the remaining 25% were female. The average weight and body mass index (BMI) was 32.3 kg (±11.8) and 20 kg/m² (±4.5), respectively. Abnormal hemoglobin levels and/or white blood cell counts were not observed. The average time of sun exposure was 6.3 h a day.

**Figure 1:**
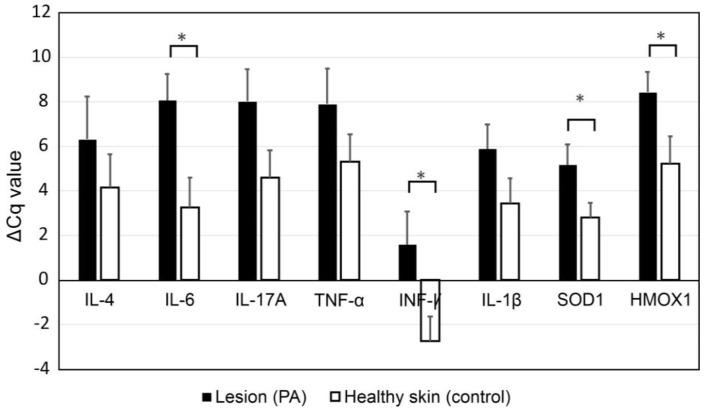
Analysis of delta Cq (ΔCq) values of the genes of interest in skin with and without lesions from children with Pytiriasis Alba. ΔCq values were obtained for genes of interest using *GAPDH* as the internal control. The obtained values were compared between skin with and without lesions (control) from the same children. Data are represented as the mean ± SE of ΔCq values from 16 participants (see Materials and Methods section for details). Significant *p*-values (*p* < 0.05) are highlighted with an asterisk.

The panel of eight genes related to oxidative stress and the inflammatory response was evaluated in 32 skin biopsies from 16 patients. Sixteen of these biopsies were from the lesion area, and 16 were from healthy skin. Figure 1 shows the results of the analysis of ΔCq values of the genes of interest in lesions and healthy areas from children with PA.

**Figure 2:**
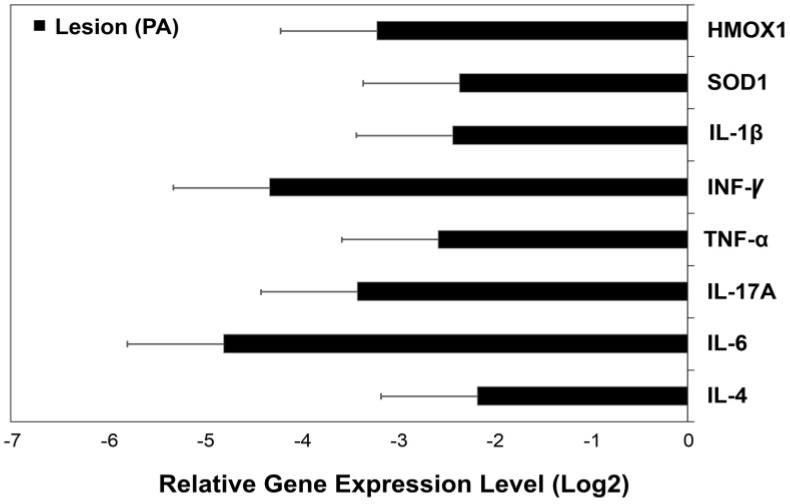
Expression levels of the genes of interest in injured skin from children with Pytiriasis Alba. Delta Cq (ΔCq) values of the genes of interest were used to calculate the relative expression level for each gene of interest by the 2^−ΔΔCq^ method, using the healthy skin data from each participant (control) as a calibrator. The mean of Log 2 of the expression level ± SE (see Materials and Methods section for details) is shown.

There were differences in ΔCq values for IL-6 (*p* = 0.012), INFγ (*p* = 0.02), SOD1 (*p* = 0.036), and HMOX1 (*p* = 0.042) between tissue with lesions and healthy skin (*p* < 0.05). Differences in ΔCq values of IL-4, IL-17A, TNFα, and IL-1β between damaged skin and control skin were not identified. Compared to healthy skin, skin with lesions showed under-expression of IL-6, INFγ, SOD1, and HMOX1, respectively (Figure 2). The range of expression for the significant genes was from −1.86 to −0.403 for IL-6, −2.34 to 1.12 for INFγ, −3.95 to −0.602 for SOD1, and −2.59 to 0.59 for HMOX1, respectively (See Figure 2).

Twenty-five per cent of skin biopsies with lesions showed over-expression of IL-6, INFγ, SOD1, and HMOX1, respectively (Supplementary Figure S1).

Table 3 displays the results of the correlation analysis of the expression levels for the genes of interest and the clinical variables. There was a positive correlation between HMOX1 expression and weight (*p* = 3.8 × 10^−3^, correlation coefficient: 0.718), height (*p* = 8.3 × 10^−3^, correlation coefficient: 0.673) and BMI (*p* = 3.5 × 10^−2^, correlation coefficient: 0.565). Strong positive correlations between the expression of IL-6 and IL-17, INFγ, IL-1B, SOD1, and HMOX1 were also observed (*p* < 0.05).

## 4. Discussion

**Figure 3:**
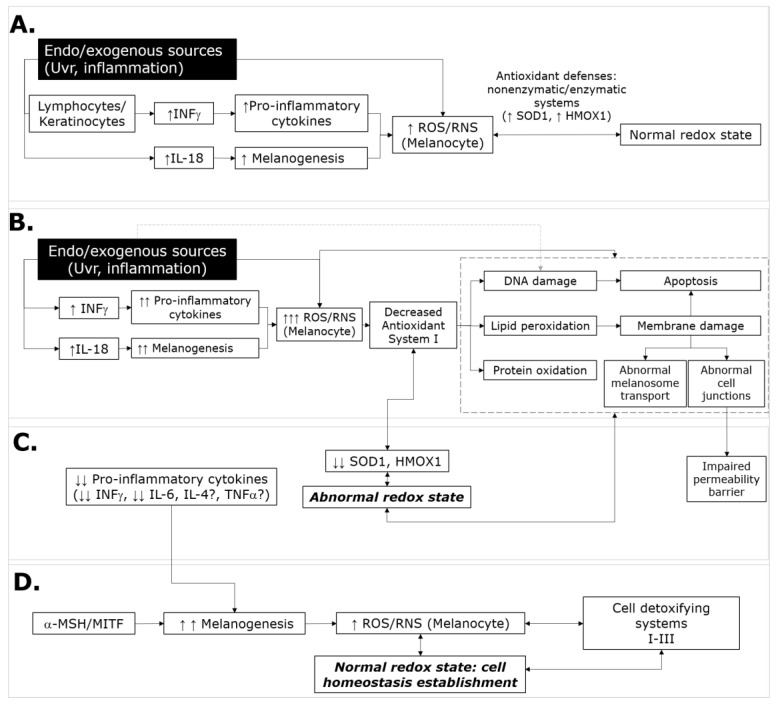
Representation response of skin to ultraviolet radiation (UVr) or other endogenous or exogenous sources of cell damage. In normal conditions (**A**), when UV light radiates the entire skin layer, it causes photodamage and induces melanogenesis [21]. Other endogenous sources of skin damage, like inflammation, induce the production of pro-inflammatory cytokines and generate ROS and RNS [11,12,21]. Melanogenesis generates ROS and RNS, inhibits pro-inflammatory cytokines, and induces pigmentation of the epidermis [22,23]. The pro-inflammatory cytokines induce the production of collagen, while the antioxidant systems combat OS, restoring the cell equilibrium [13,21,24]. When UVr falls on dysfunctional or immature skin (**B**) or occurs chronically, the skin is photodamaged, and type I antioxidant systems gradually weaken. Skin damage generates ROS and RNS, as well as pro-inflammatory cytokines—mainly IL-6 and INF-γ. ROS and RNS induce lipid peroxidation, and this process, in turn, increases the oxidative/nitrosative stress state, causing apoptosis, affecting melanogenesis and the transport of melanosomes. These events may produce hypopigmentation, hyperkeratosis, and leucocyte infiltrations [34,35,36]. Gradually (**C**), the UVr depletes cell antioxidants and reduces the efficiency of the skin’s antioxidant systems [26]. OS causes failures in the regulation of melanocyte-inducing transcription factor (MITF), sensitizing the melanocytes to OS, which leads to cell death [11,27] (**D**). Physiological basal pigmentation may occur with the suppression of immune mediators, such as INF-γ, and cytokines, such as IL-6, IL-4, and TNF-α, which are also known to downregulate melanogenesis in melanocytes [18]. Alpha-melanocyte-stimulating hormone (α-MSH) increases the expression, de novo synthesis, and activation of tyrosinase [12,29,30]. α-MSH stimulates the dendricity of melanocytes and protects them from superoxide radicals. The production of new melanin can repair the damage caused by ROS in apoptotic tissue [9,18,31,32]. *ROS*: reactive oxygen species; *RNS*: reactive nitrogen species; *AUVr*: ultraviolet A radiation; *BUVr*: ultraviolet B radiation; *INF-γ*: interferon-gamma; *IL-18:* interleukin 18 *IL-6*: interleukin 6; *α-MSH:* alpha-melanocyte-stimulating hormone.

PA is a benign and self-limited dermatological condition that is characterized by the appearance of hypopigmented macules with undefined edges in photo-exposed sites [5]. The stratified architecture of the skin, and its antioxidant systems and melanogenesis capacity, are the major defense mechanisms against ultraviolet radiation (UVr) [18]. Considering that UV exposition of the skin promotes an acute inflammatory response and an increase of OS in other skin diseases [8,9,14,19,20], we evaluated the expression levels of inflammatory and OS-related genes in skin biopsies and their association with PA. Our results showed differences in the ΔCq values of pro-inflammatory cytokine genes (*IL-6* and *INFγ*) and antioxidant-related genes (*SOD1* and *HMOX1*) between skin biopsies with and without lesions. Compared with healthy skin, the expression levels of these genes were predominantly downregulated in lesions. Currently, there are no studies that have evaluated the expression of cytokines and/or OS-related genes in PA. However, in order to explain our results, in Figure 3, our actual knowledge regarding the skin response to exogenous and/or endogenous sources of cell damage was summarized, and our results were also integrated as follows: in normal conditions, when UV light radiates the entire skin layer, it causes photodamage and induces melanogenesis [21] (Figure 3A). Similar to UVr, endogenous sources of skin damage, such as inflammation, induce the production of pro-inflammatory cytokines and generate ROS and RNS [11,12,21]. Melanogenesis, in turn, generates ROS and RNS, and induces pigmentation of the epidermis [22,23]. The pro-inflammatory cytokines may induce the production of collagen, while antioxidant systems combat OS, restoring the cell equilibrium [13,21,24]. When UVr falls on dysfunctional or immature skin, or irradiates chronically (Figure 3B), it produces photodamage and initiates the depletion of type I antioxidant systems. Photodamage induces ROS and RNS, as well as pro-inflammatory cytokines—mainly IL-6 and INF-γ. ROS and RNS induce lipid peroxidation, and this process, in turn, increases the oxidative/nitrosative stress state, causing several cell alterations, including protein oxidation, damage to the cell membrane and DNA, alterations in transport, and consequently, apoptosis. It has been reported that high concentrations of hydrogen peroxide and NO may also inhibit type II antioxidant systems [13,23,24]. Therefore, both pro-inflammatory cytokines and ROS/NOS production generate a persistent OS environment [18]. During the acute process, activation of Th1 lymphocytes and/or production of large amounts of INFγ by keratinocytes may activate macrophages [12,18]. Macrophages release ROS, NO, and pro-inflammatory cytokines, promoting leukocyte extravasation [2,12,25]. This process is in agreement with the perivascular lymphocytic infiltrates occasionally seen in the dermis of patients with PA [7], and it may also explain the observed over-expression of pro-inflammatory cytokines (INF-γ and IL-6) and OS-related genes (SOD1 and HMOX1) in the lesions in 25% of the participants in our study. When IFNγ is present, it stimulates CD4 and CD8 cells, which lyse various cell types, such as keratinocytes [11,12], and triggers the expression of NADPH oxidases (NOX4, and NOX1), with concomitant elevation of ROS during the acute inflammatory processes. The expression of IFNγ may produce a hypopigmentation phenotype and stop the maturation of the melanosome in stages I and II, when it still has no pigment [11,13,26]. Over time, UVr depletes cell antioxidants and reduces the efficiency of the skin’s antioxidant systems (Figure 3C) [26]. It has been reported that OS causes failures in the regulation of melanocyte inducing transcription factor (MITF), which sensitizes melanocytes to OS and leads to their death [11,27]. Furthermore, the recurrent inflammatory environment causes epidermal cells to enter a hypermetabolic state [28]. Likewise, the excessive production of metabolites and oxidizing agents of melanocyte leads to autolysis [3]. These facts may explain in part the presence of hyperkeratosis, observed in histological studies [7].

Physiological mediators that reinstate basal pigmentation are difficult to deduce because of their transient expression [18]. However, repigmentation of the lesions likely occurs with the suppression of immune mediators, such as INF-γ, and cytokines, such as IL-6, IL-4, and TNF-α, which are also known to downregulate melanogenesis in melanocytes [18] (Figure 3D). This hypothesis is supported by our results, in which the expression of these mediators was positive and strongly correlated. Likewise, the participation of alpha-melanocyte-stimulating hormone (α-MSH) in the establishment of the normal redox state and cell homeostasis is supported, because it may increase the expression, synthesis de novo, and activation of tyrosinase (a mediator of melanin production) [12,29,30]. Therefore, α-MSH stimulates the dendricity of melanocytes, and protects them from superoxide radicals. The production of new melanin can repair the damage caused by ROS in apoptotic tissue [9,18,31,32]. These changes may have an effect on the restoration of the permeability barrier, which is significantly impaired in PA [33].

In spite of the fact that additional studies must be completed to reproduce our results, we consider that the PA phase is critical for the selection of treatment, which may be focused on anti-inflammatory medication as the first step and then on topical antioxidants that can lead to the repigmentation of damaged areas [8,25,34]. Because recurrent exposure to UVr can prolong hypochromia for over 10 years, the prevention of UVr exposition is indicated to decrease how long a patient has lesions [4,5,8,24], and to avoid their recurrence.

Finally, some study limitations must be highlighted. For instance, the number of participants included in the study was small, and therefore additional studies must be completed to reproduce our results. In the same sense, due to the small number of subjects, with significant genes differentially expressed, additional statistical comparisons, such as over/under-expression and features of lesions, could not be carried out. These important issues should be considered in future experimental approaches to the study of the molecular pathophysiology of PA.

## 5. Conclusions

Compared with healthy skin, *HMOX1*, *SOD1*, *IL6* and *IFNγ* were under-expressed in the skin lesions of patients with PA. However, 25% of biopsies with PA-related lesions showed over-expression of these four genes, and strong positive correlations between their expressions were also identified. As such, we propose the presence of molecular stages of PA, defined according to the over-expression (first stage) or under-expression (second stage) of *HMOX1*, *SOD1*, *IL-6*, and *IFNγ* in abnormal tissue. These findings may have implications for the selection of treatments for PA-related lesions.

## Figures and Tables

**Table 1:** General information of the oligonucleotides used in the study for the qRT-PCR assays.

Gene Symbol	GenBank Number	Primer Sequence (5′-3′)	Tm (°C)	Product Size (bp)
IL-4	NM_000589	F: GACATCTTTGCTGCCTCCAA	60	128
		R: GTGCGACTGCACAGCAGTT		
IL-6	NM_000600	F: CCCTGAGAAAGGAGACATGT	60	111
		R: TGAAAAAGATGGATGCTTCCAA		
IL-17A	NM_002190	F: TGGAATCTCCACCGCAATGA	60	116
		R: GTGGACTACCACATGAACTC		
TNF-α	NM_000594	F: CAGGCAGTCAGATCATCTTC	60	121
		R: CCAATGCCCTCCTGGCCA		
IFN-γ	NM_000619	F: AGGAAGACATGAATGTCAAGTT	60	108
		R: GAATGTCCAACGCAAAGCAAT		
IL-1β	NM_000576	F: GGAGCAACAAGTGGTGTTCT	60	116
		R: ACCTGTCCTGCGTGTTGAAA		
SOD1	NM_000454	F: GAGGCATGTTGGAGACTTGG	60.5	205
		R: ACAAGCCAAACGACTTCCAG		
HMOX1	NM_002133	F: GCTCAACATCCAGCTCTTTGA	60.5	196
		R: TGTAAGGACCCATCGGAGAA		
GAPDH	NM_002046	F: GAGTCAACGGATTTGGTCGT	60.1	214

**Table 2:** General characteristics of the study population.

Variable	Patients (n = 16)
Gender	
Male, n (%)	12 (75)
Female, n (%)	4 (25)
Age (years)	8.9 ± 3.1
Weight (kg)	32.3 ± 11.8
Height (cm)	125.4 ± 14.3
Body mass index (kg/m²)	20.0 ± 4.5
Hemoglobin (mg/dL)	13.4 ± 1.5
Leucocytes (10^3^/μL)	7.3 ± 2.5
Lymphocytes (10^3^/μL)	3103.8 ± 1932.3
Neutrophils (10^3^/μL)	3674.4 ± 2406.5
Creatinine (mg/dL)	0.41 ± 0.10
Sun exposure (h/day)	6.31 ± 1.44

Data are presented as the frequency and percentage, or as the mean ± standard deviation.

**Table 3:** Correlation test between genes and clinical variables.

Variable 1	Variable 2	Correlation Coefficient	*p*-Value
IL-4	HMOX1	0.611	3.5 × 10^−^^2^
IL-6	IL-17	0.924	3.1 × 10^−^^7^
	INF-γ	0.955	9.3 × 10^−^^9^
	IL-1B	0.915	6.8 × 10^−^^7^
	SOD1	0.944	4.2 × 10^−^^8^
	HMOX1	0.564	3.6 × 10^−^^2^
IL-17	INF-γ	0.936	9.6 × 10^−^^8^
	IL-1B	0.953	1.2 × 10^−^^8^
	SOD1	0.892	3.5 × 10^−^^6^
TNF-α	HMOX1	0.769	1.3 × 10^−^^3^
INF-γ	IL-1B	0.854	2.5 × 10^−^^5^
	SOD1	0.922	3.8 × 10^−^^7^
	HMOX1	0.549	4.2 × 10^−^^2^
IL-1B	SOD1	0.857	2.3 × 10^−^^5^
SOD1	HMOX1	0.567	3.5 × 10^−^^2^
HMOX1	Weight	0.718	3.8 × 10^−^^3^
	Height	0.673	8.3 × 10^−^^3^
	Body mass index	0.565	3.5 × 10^−^^2^

## Supplementary Materials

The following are available online at https://www.mdpi.com/1010-660X/56/7/359/s1, Figure S1: Representation of the individual relative expression of genes, showing differences between skin with and without lesions.
