# Supplementary material for: Expression Levels of Inflammatory and Oxidative Stress-Related Genes in Skin Biopsies and Their Association with Pityriasis Alba
Source: Medicina (Kaunas). 2020 Jul 17;56(7):359. doi: 10.3390/medicina56070359 (PMC7404726; doi:10.3390/medicina56070359)
Supplement: Supplementary file 1 [file medicina-56-00359-s001.pdf]

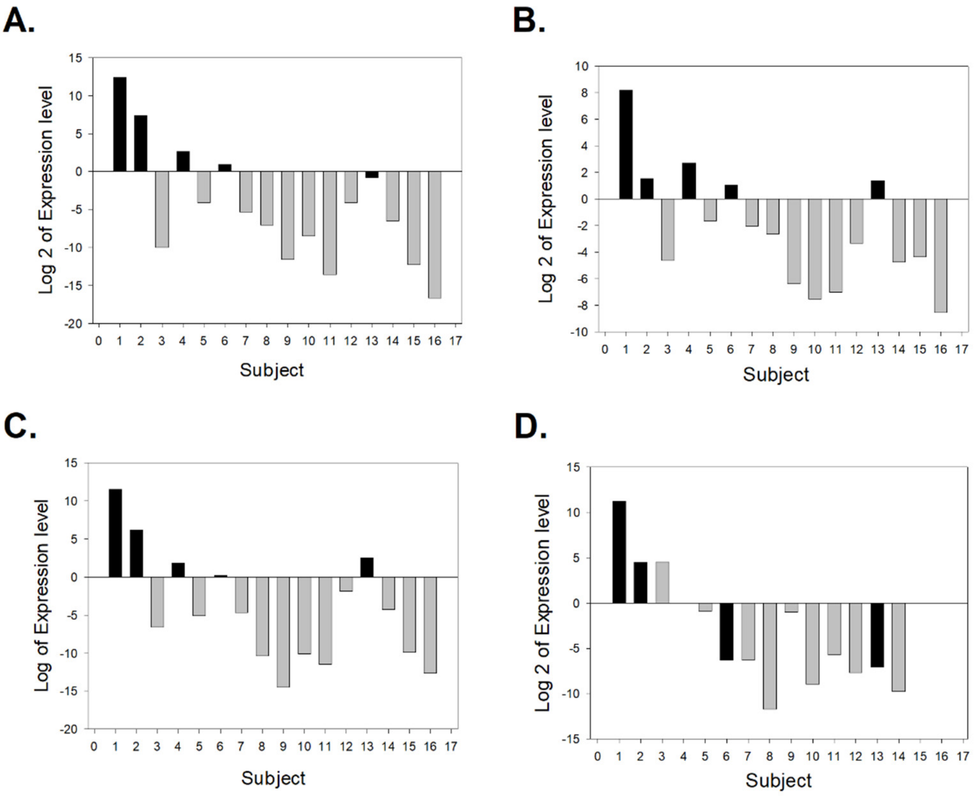

Figure S1. Representation of the individual relative expression of genes with differences between skin with and without lesion. Each plot represents the Log 2 of relative gene expression of each skin biopsy from each study participant: (**A**, **C**) IL-6 and IFNγ, and (**B**, **D**) SOD-1 and HMOX-1, respectively. In dark bars, the patients 1, 2, 4, 6, and 13 are highlighted.
